# A Case of Clinically Suspected Kounis Syndrome Associated with Platelet Transfusion during Thoracoscopic Right Lower Lobectomy

**DOI:** 10.70352/scrj.cr.26-0204

**Published:** 2026-06-23

**Authors:** Tomohiro Baba, Hidetoshi Inokawa, Masanori Okada, Riki Okita

**Affiliations:** 1 Division of Thoracic Surgery, National Hospital Organization Yamaguchi Ube Medical Center, Ube, Yamaguchi, Japan; 2 Department of General Thoracic Surgery and Breast and Endocrinological Surgery, Okayama University Graduate School of Medicine, Dentistry and Pharmaceutical Sciences, Okayama, Okayama, Japan

**Keywords:** Kounis syndrome, platelet transfusion, anaphylaxis, intraoperative, pulmonary lobectomy

## Abstract

**INTRODUCTION:**

Kounis syndrome is an acute coronary syndrome associated with allergic or hypersensitivity reactions and is characterized by the concurrent occurrence of allergic manifestations and coronary artery spasm or myocardial ischemia. Transfusion-related cases, particularly those associated with platelet transfusion, are extremely rare. We report a case of clinically suspected intraoperative Kounis syndrome associated with platelet transfusion during thoracoscopic right lower lobectomy.

**CASE PRESENTATION:**

A 70-year-old man with chronic hepatitis C–associated thrombocytopenia underwent thoracoscopic right lower lobectomy for primary lung cancer. Prophylactic antibiotic administration was uneventful. Irradiated platelet concentrate was transfused intraoperatively as planned. Three minutes after completion of the platelet concentrate transfusion, the heart rate increased and hypotension developed, accompanied by flushing of the face and upper extremities. Phenylephrine and ephedrine were administered without improvement, and anaphylactic shock associated with platelet transfusion was suspected. Adrenaline, antihistamines, and methylprednisolone were administered. Shortly after a continuous intravenous adrenaline infusion was started, ST-segment depression in lead II was noted on the intraoperative electrocardiogram (ECG) monitor, and review of the ECG record confirmed that this change had been present since the onset of the hemodynamic deterioration. Cardiac biomarkers, including troponin T, showed no elevation. Because hemodynamics stabilized under continuous intravenous adrenaline infusion and oxygenation and ventilation remained stable, the surgery was continued and completed thoracoscopically. Postoperative 12-lead electrocardiography showed ST-segment depression in leads II, III, and augmented vector foot, which was resolved by POD 1, without echocardiographic wall motion abnormalities. The postoperative course was uneventful.

**CONCLUSIONS:**

The clinical course and electrocardiographic findings were clinically suggestive of Type I Kounis syndrome. However, because direct coronary assessment, such as coronary angiography, was not performed, a definitive diagnosis could not be established. Under general anesthesia, subjective symptoms cannot be reliably assessed, and hemodynamic instability or ECG abnormalities may be the only clues. Therefore, when allergic reactions and ischemic ECG changes occur intraoperatively, Kounis syndrome should be considered in the differential diagnosis. Platelet transfusion may be associated with intraoperative clinically suspected Kounis syndrome. Prompt recognition, hemodynamic stabilization, and close collaboration between anesthesiologists and surgeons, with strict hemodynamic and electrocardiographic monitoring, are essential for appropriate perioperative management.

## Abbreviations

ECG
: electrocardiogram
LVEF
: left ventricular ejection fraction
S-1
: tegafur–gimeracil–oteracil potassium

## INTRODUCTION

Kounis syndrome is an acute coronary syndrome associated with allergic or hypersensitivity reactions, first proposed by Kounis and Zavras in 1991.^1)^ It is characterized by the simultaneous occurrence of allergic manifestations and coronary artery spasm or myocardial ischemia. Although this condition has been reported in association with triggers, including drugs, contrast media, foods, and insect stings, transfusion-related cases, particularly those associated with platelet transfusion, are exceedingly rare.^2,3)^

Intraoperative occurrence is uncommon but can result in life-threatening complications such as hemodynamic instability and cardiac arrest.^4)^ Moreover, this condition remains underrecognized and may be underdiagnosed.^5)^

Herein, we report a rare case of clinically suspected Kounis syndrome associated with platelet transfusion during thoracoscopic right lower lobectomy and discuss its clinical features and perioperative considerations.

## CASE PRESENTATION

A 70-year-old man was referred to the Division of Thoracic Surgery, National Hospital Organization Yamaguchi Ube Medical Center for surgical treatment of a right lower lobe lung tumor. He had chronic hepatitis C–associated thrombocytopenia and a history of gastric cancer treated surgically, followed by postoperative chemotherapy with S-1.

CT revealed a 4-cm mass in the right lower lobe. An initial CT-guided biopsy did not yield a definitive diagnosis, and the patient was followed up. A follow-up CT scan the following year revealed that the mass had enlarged to 5.5 cm, and a repeat CT-guided biopsy confirmed primary lung adenocarcinoma. PET/CT showed fluorodeoxyglucose uptake in the tumor (maximum standardized uptake value, 10–11) without evidence of lymph node or distant metastasis. The patient was diagnosed with primary lung adenocarcinoma of the right lower lobe (cT3N0M0, stage IIB) and was referred to the Division of Thoracic Surgery, National Hospital Organization Yamaguchi Ube Medical Center for surgical treatment.

He had a 50–pack-year smoking history, and his regular medications included vonoprazan fumarate and S-1. He had no history of blood transfusion, including red blood cell or platelet transfusion. On admission, his performance status was 0. Physical examination revealed no remarkable findings. Laboratory tests demonstrated thrombocytopenia and impaired liver function corresponding to Child–Pugh class B (score, 7). Staging contrast-enhanced CT revealed a 5.5-cm irregular solid mass in the right lower lobe, with no significant lymphadenopathy or distant metastasis (**Fig. 1**). A 12-lead ECG showed no significant abnormalities, and transthoracic echocardiography demonstrated preserved LVEF (72%) with no regional wall motion abnormalities or significant valvular disease.

**Fig. 1:**
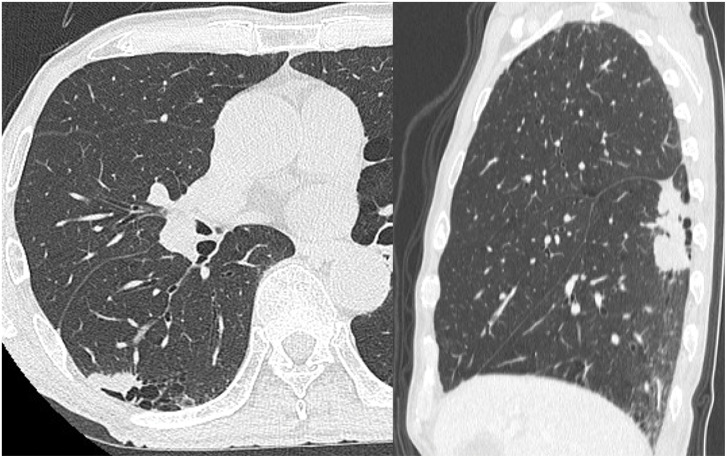
Chest CT findings. CT showed an irregular solid mass in the right lower lobe measuring approximately 5.5 cm in maximum diameter.

S-1 had been discontinued 2 weeks before surgery; however, the platelet count remained low at 6.3 × 10^4^/μL 2 days before surgery. Following consultation with the Department of Anesthesiology and Resuscitology, Okayama University Graduate School of Medicine, Dentistry and Pharmaceutical Sciences, it was decided to proceed with surgery with planned transfusion of 10 units of platelet concentrate.

Under general anesthesia with 1-lung ventilation, the patient was placed in the left lateral decubitus position, and thoracoscopic right lower lobectomy with lymph node dissection was initiated. Anesthesia was induced with fentanyl, propofol, and rocuronium without incident. Prophylactic cefazolin was administered uneventfully, and hemodynamics remained stable for 10 min after completion of the cefazolin infusion. Transfusion of 10 units of irradiated platelet concentrate was then started.

Three minutes after completion of the platelet concentrate transfusion, the heart rate increased and hypotension developed. Although assessment of the trunk was limited because it was covered by sterile drapes, cutaneous flushing was noted on the face and upper extremities. Despite administration of phenylephrine and ephedrine, hemodynamic improvement was insufficient, and anaphylactic shock associated with platelet transfusion was suspected. As initial treatment, intramuscular adrenaline (0.3 mg) and antihistamines were administered, resulting in transient hemodynamic improvement. Because the effect of the intramuscular dose was short-lived, continuous intravenous adrenaline infusion (0.02 μg/kg/min) and intravenous methylprednisolone (250 mg) were initiated (**Fig. 2**).

**Fig. 2:**
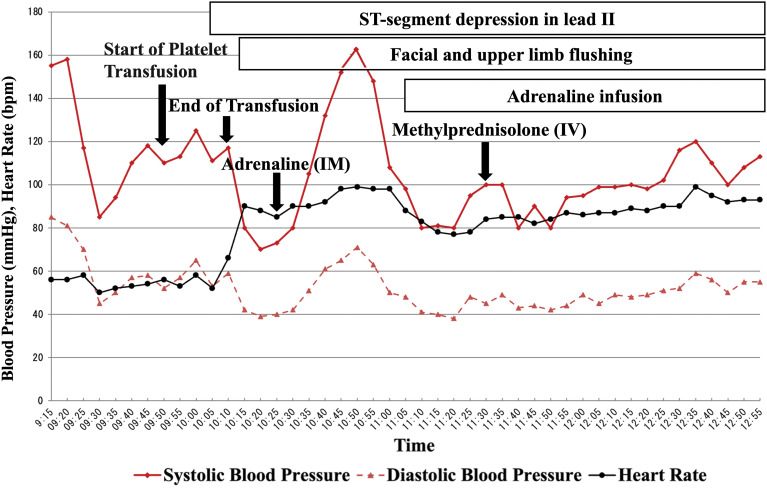
Intraoperative clinical course. Three minutes after completion of platelet concentrate transfusion, hypotension and an increased heart rate with flushing developed, accompanied by ST-segment depression in lead II on intraoperative ECG monitoring. Intramuscular adrenaline (0.3 mg) produced transient hemodynamic improvement. Because the effect of the intramuscular dose was short-lived, continuous intravenous adrenaline infusion (0.02 μg/kg/min) and intravenous methylprednisolone (250 mg) were initiated. Hemodynamics stabilized thereafter, and the thoracoscopic procedure was completed under strict hemodynamic and electrocardiographic monitoring. ECG, electrocardiogram; IM, intramuscular; IV, intravenous

Shortly after a continuous intravenous adrenaline infusion was started, the anesthesiologist noticed ST-segment depression in lead II on the ECG monitor. Review of the intraoperative ECG record confirmed that this change had been present since the onset of the hemodynamic deterioration, raising suspicion of Kounis syndrome associated with an allergic reaction. Cardiac biomarkers, including troponin T, were not elevated.

Discontinuation of the procedure was considered. However, after discussion with the anesthesiologist, the operation was continued because oxygenation and ventilation remained stable, bronchospasm was not observed, and hemodynamics was maintained under continuous intravenous adrenaline infusion. The procedure was completed thoracoscopically as planned. The operative time was 249 min, and the estimated blood loss was 20 mL.

Postoperatively, the patient was placed in the supine position. Transthoracic echocardiography showed no wall motion abnormalities, with an LVEF of 70%. A 12-lead ECG demonstrated persistent ST-segment depression in leads II, III, and augmented vector foot, along with T-wave inversion in lead V3 (**Fig. 3**), although the flushing had resolved. A central venous catheter was placed via the right internal jugular vein. After emergence from anesthesia, the patient was extubated in the operating room and transferred to the ward with stable hemodynamic and respiratory status while on continuous intravenous adrenaline infusion.

**Fig. 3:**
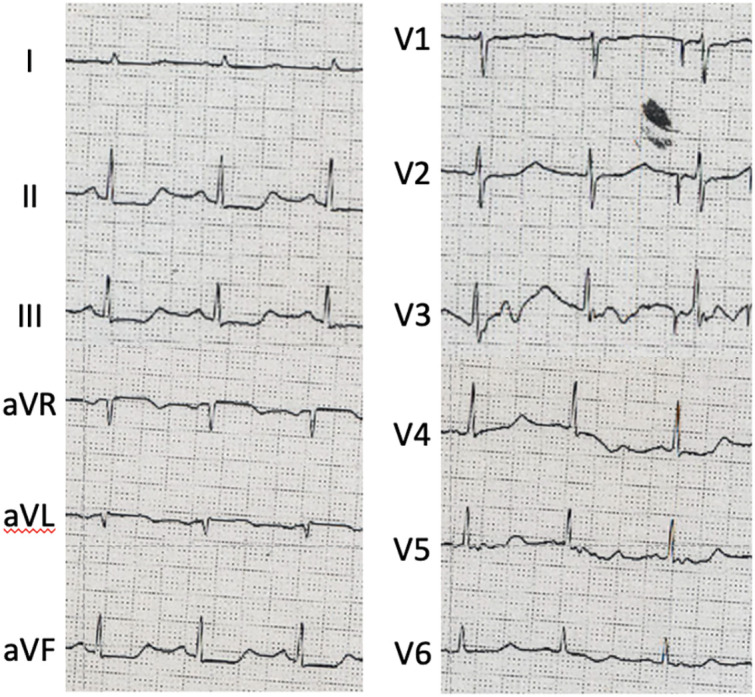
Postoperative 12-lead ECG. A 12-lead ECG obtained immediately after surgery showed ST-segment depression in leads II, III, and aVF, along with T-wave inversion in lead V3. aVF, augmented vector foot: aVL, augmented vector left; aVR, augmented vector right; ECG, electrocardiogram

Adrenaline was tapered and discontinued by the morning of POD 1. Follow-up 12-lead ECG on POD 1 showed resolution of the ST-segment depression and T-wave inversion (**Fig. 4**). The central venous catheter and chest drain were removed on POD 2 and POD 7, respectively, and the patient was discharged home on POD 14.

**Fig. 4:**
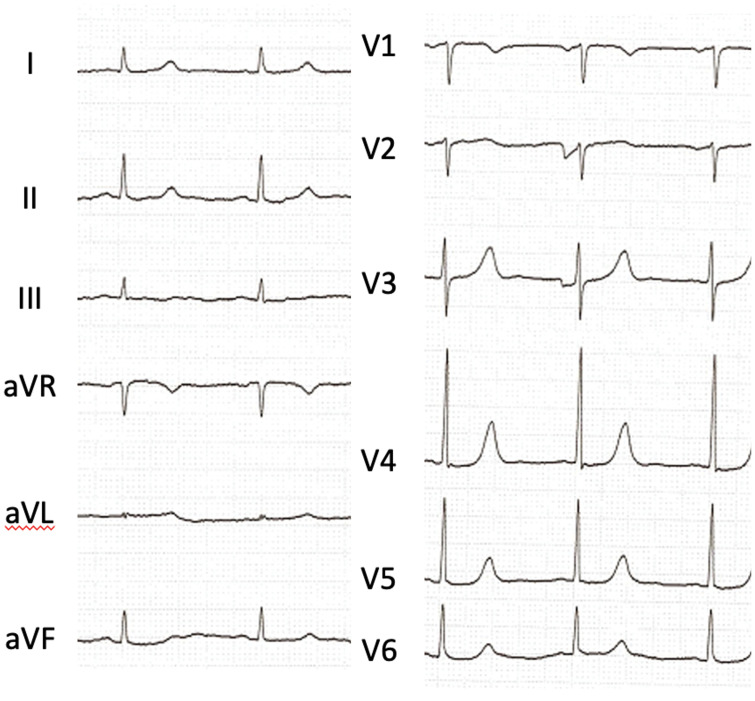
Follow-up 12-lead ECG on POD 1. A 12-lead ECG on POD 1 showed resolution of the ST-segment depression and T-wave inversion. aVF, augmented vector foot: aVL, augmented vector left; aVR, augmented vector right; ECG, electrocardiogram

Final pathology revealed squamous cell carcinoma of the right lower lobe (pT2bN0M0, pStage IIA), differing from the preoperative biopsy diagnosis of adenocarcinoma. No recurrence has been observed to date.

## DISCUSSION

Kounis syndrome is categorized into 3 types: Type I involves coronary artery spasm in patients without underlying coronary artery disease; Type II occurs in patients with pre-existing atherosclerotic lesions; and Type III is associated with coronary stent thrombosis.^1)^ In our case, the patient had no history of coronary artery disease, exhibited transient ST-segment depression without elevation of cardiac biomarkers, and showed no wall motion abnormalities on transthoracic echocardiography. Collectively, these findings were clinically suggestive of Type I Kounis syndrome.

A characteristic feature of this case was the intraoperative onset of clinically suspected Kounis syndrome associated with platelet transfusion during thoracoscopic pulmonary resection. Transfusion-related Kounis syndrome is extremely rare, and reports specifically involving platelet transfusion are particularly limited. To our knowledge, only 1 such case has been previously documented.^3)^ Therefore, the present case represents a rare additional report of clinically suspected Kounis syndrome associated with platelet transfusion.

In this patient, no hemodynamic changes were observed after prophylactic antibiotic administration, whereas an increased heart rate, hypotension, and cutaneous flushing occurred 3 min after completion of the platelet concentrate transfusion. Review of the intraoperative ECG record also revealed ST-segment depression at the onset of these changes. This temporal relationship suggests an association between platelet transfusion and the onset of clinically suspected Kounis syndrome. Although allergic reactions to other perioperative agents, such as cefazolin and anesthetic drugs, cannot be completely excluded, no worsening suggestive of anaphylaxis was observed after intraoperative redosing of cefazolin or administration of neuromuscular blocking agents. Taken together, platelet transfusion was considered the most likely trigger in this case.

In the present case, the event was initially managed as suspected anaphylactic shock associated with platelet transfusion, and hemodynamic stabilization was achieved with low-dose continuous intravenous adrenaline infusion. When Kounis syndrome was clinically suspected intraoperatively, a coronary vasodilator such as nicorandil was also considered. However, because coronary vasodilators carried a risk of further hypotension in this setting, priority was given to maintaining hemodynamic stability with low-dose adrenaline infusion. Nevertheless, because adrenaline may theoretically aggravate myocardial ischemia or coronary vasospasm by increasing myocardial oxygen demand, the infusion was continued only with careful monitoring of the ECG and hemodynamic status.

Although the clinical course was highly suggestive of Type I Kounis syndrome, anaphylactic shock associated with platelet transfusion alone, along with secondary transient ischemic ECG changes due to hypotension, could not be completely excluded. In addition, serum histamine and tryptase levels were not measured at the time of the event, and formal allergy testing and direct coronary assessment were not performed. Therefore, the diagnosis remained clinical and circumstantial.

Perioperatively, multiple drugs and blood products may trigger allergic reactions, and Kounis syndrome should be considered when sudden hemodynamic deterioration occurs after administration of new drugs or blood products.^2,6)^ In particular, under general anesthesia, subjective symptoms cannot be reliably assessed, and hemodynamic instability or ECG abnormalities may be the initial manifestations, making intraoperative diagnosis challenging.^6,7)^

Management of Kounis syndrome requires simultaneous treatment of the allergic reaction and circulatory management as acute coronary syndrome. Immediate discontinuation of the causative antigen is the highest priority, and prompt initial treatment, including adrenaline administration, is required in cases of hemodynamic instability. Because coronary artery spasm may be involved, the concomitant use of steroids and antihistamines, as well as nitrates and calcium channel blockers, has been recommended.^5,8)^ Although adrenaline is the first-line agent for anaphylaxis, its α-adrenergic effects may exacerbate coronary artery spasm; therefore, careful hemodynamic and electrocardiographic monitoring is required in perioperative cases under general anesthesia.^4)^ In the present case, low-dose continuous intravenous adrenaline infusion enabled stable hemodynamic control under strict monitoring, while oxygenation and ventilation remained stable.

When Kounis syndrome is suspected intraoperatively, a key decision is whether to continue or abort the surgical procedure while initiating treatment. In the present case, the decision to continue surgery was based primarily on immediate perioperative factors indicating that the acute reaction was controllable under strict intraoperative management. Specifically, blood pressure was maintained with low-dose continuous intravenous adrenaline infusion, while oxygenation and ventilation remained stable and bronchospasm was not observed, suggesting that the acute reaction was controllable under strict intraoperative management. In addition, because the patient had resectable lung cancer, delaying surgery until formal allergy testing could be completed might have imposed a clinical disadvantage. Furthermore, if platelet transfusion had indeed been the trigger, recurrence during a subsequent operation requiring transfusion could not be excluded. These factors were considered supportive rather than primary reasons for continuing the procedure. Therefore, after careful discussion with the anesthesiologist and comprehensive assessment of the overall clinical condition, the operation was continued and completed thoracoscopically. However, perioperative Kounis syndrome progressing to cardiac arrest has been reported,^4,6)^ and careful assessment of circulatory and respiratory status is essential when deciding whether to continue or abort surgery. Conversely, successful completion of surgery under strict management has also been reported in cases occurring under general anesthesia.^7)^ In such situations, discontinuation of the procedure should always remain an option, and the decision must be individualized.

## CONCLUSIONS

This case represents a rare instance of clinically suspected Kounis syndrome associated with platelet transfusion during thoracoscopic pulmonary resection. When Kounis syndrome is suspected intraoperatively, close collaboration between anesthesiologists and surgeons is essential for prompt recognition and hemodynamic stabilization. Careful assessment of circulatory and respiratory stability is required when determining whether surgery can be safely continued.

## DECLARATIONS

### Availability of data and materials

The datasets supporting the conclusions of this article are included within the article.
